# Clinical Significance of Vulvar Condyloma as a Marker of Concurrent High-Risk Cervical HPV Infection and Abnormal Cervical Cytology

**DOI:** 10.3390/diagnostics16152439

**Published:** 2026-08-02

**Authors:** Eda Güner Özen, Süleyman Özen, Özgün Akbaş, Ahkam Göksel Kanmaz, Yaşam Kemal Akpak

**Affiliations:** 1 Department of Obstetrics and Gynecology, Izmir City Hospital, 35540 Izmir, Türkiye; ozgunakbas1@gmail.com (Ö.A.); drgokselkanmaz@gmail.com (A.G.K.); drykakpak@gmail.com (Y.K.A.); 2 Department of Gynecologic Oncology, Izmir City Hospital, 35540 Izmir, Türkiye; suleymanozen90@gmail.com; 3 Izmir Faculty of Medicine, University of Health Sciences, 35540 Izmir, Türkiye

**Keywords:** vulvar condyloma, human papillomavirus, cervical cytology, cervical dysplasia, HPV16, HPV18, cervical intraepithelial neoplasia

## Abstract

**Background/Objectives:** Human papillomavirus (HPV) infection is the principal etiological factor in cervical cancer and its precursor lesions. Although vulvar condyloma is typically associated with low-risk HPV types, high-risk HPV genotypes may coexist. This study evaluated the association between vulvar condyloma, cervical cytological abnormalities, high-risk HPV positivity, and cervical histopathological outcomes. **Methods:** This retrospective observational study included 407 women evaluated at the Department of Obstetrics and Gynecology, Izmir City Hospital, between January 2025 and January 2026. Patients underwent cervical cytology, HPV testing, vulvar examination, and colposcopic biopsy when clinically indicated. Patients were categorized according to the presence or absence of histopathologically confirmed vulvar condyloma. Logistic regression analyses were performed to identify factors associated with vulvar condyloma. **Results:** Vulvar condyloma was identified in 180 patients (44.2%). Abnormal cervical cytology was recorded in 66/180 patients (36.7%) with vulvar condyloma and 41/227 patients (18.1%) without condyloma (*p* < 0.001). High-risk HPV positivity was observed in 104/180 (57.8%) and 86/227 (37.9%) patients, respectively (*p* < 0.001). In multivariate analysis, adjusted ORs were 2.30 for HPV16 positivity (95% CI 1.36–3.89, *p* = 0.002), 3.29 for HPV18 positivity (95% CI 1.40–7.73, *p* = 0.006), and 2.01 for abnormal cervical cytology (95% CI 1.24–3.25, *p* = 0.005). Recorded CIN2+ was 15/180 (8.3%) in the condyloma-positive group and 7/227 (3.1%) in the condyloma-negative group in the overall cohort; in the biopsy-restricted analysis, CIN2+ was 15/82 (18.3%) and 7/41 (17.1%), respectively. **Conclusions:** Vulvar condyloma was independently associated with abnormal cervical cytology and high-risk HPV infection, particularly HPV16/18 positivity. These robust associations suggest that vulvar condyloma may represent a clinically visible marker of concurrent cervical HPV-related disease. Careful cervical evaluation is warranted in women presenting with vulvar condyloma, whereas CIN2+ findings should be interpreted cautiously until confirmed in larger prospective cohorts with standardized histopathological verification.

## 1. Introduction

Human papillomavirus (HPV) infection is one of the most common sexually transmitted infections worldwide and represents the primary etiological factor in cervical cancer and its precursor lesions. Persistent infection with high-risk HPV genotypes, particularly HPV16 and HPV18, plays a central role in the development of cervical intraepithelial neoplasia (CIN) and invasive cervical cancer [1,2].

HPV infection can manifest in various clinical forms within the lower anogenital tract. One of the most common benign manifestations is condyloma acuminatum, also known as genital warts, which are predominantly associated with low-risk HPV genotypes such as HPV6 and HPV11 [3]. However, several studies have demonstrated that individuals presenting with clinically apparent genital warts may simultaneously harbor high-risk HPV genotypes, suggesting that multiple HPV types may coexist within the same host [4]. Because the cervix, vulva, vagina, and anus form a continuous epithelial environment susceptible to HPV infection, viral involvement may occur at multiple anatomical sites [5].

Previous epidemiological studies have suggested that women presenting with genital warts may have a higher prevalence of cervical HPV infection and cervical cytological abnormalities compared with the general population [6]. Nevertheless, the relationship between vulvar condyloma, HPV genotype distribution, cervical cytological abnormalities, and histopathologically confirmed cervical dysplasia remains incompletely understood.

Recent investigations have emphasized that cervical risk stratification may be strengthened by incorporating molecular features beyond binary HPV positivity. In a real-world series of cervical specimens, higher HPV viral loads and multiple HPV infections were more frequently observed in high-grade CIN [7]. A subsequent study demonstrated the feasibility of integrating HPV genotyping and viral-load assessment with histopathological evaluation of formalin-fixed, paraffin-embedded cervical tissue to improve diagnostic precision [8]. These findings support a multidimensional approach in which the clinical presentation of HPV-related lower genital tract disease is interpreted alongside cervical cytology, genotype-specific HPV results, and, when indicated, histopathology.

Over the past decade, prophylactic HPV vaccination has substantially altered the epidemiology of HPV-related diseases in countries where national vaccination programs have been implemented. Large population-based studies have demonstrated significant reductions in both genital warts and high-grade cervical intraepithelial neoplasia following the introduction of HPV vaccination programs [9]. Population-level analyses have also shown marked decreases in the incidence of anogenital warts and CIN2+ lesions among vaccinated cohorts, highlighting the effectiveness of vaccination against HPV types responsible for both benign and premalignant disease [10].

Despite these global advances, HPV vaccination has not yet been incorporated into the national immunization program in Türkiye, and vaccine uptake remains limited. As a result, HPV-related diseases, including genital warts and cervical epithelial abnormalities, continue to represent a substantial component of routine gynecological practice. In this setting, the epidemiological characteristics of HPV infection are less influenced by vaccine-induced changes in genotype distribution, allowing evaluation of disease patterns that more closely reflect the natural history of HPV infection. Consequently, investigating the relationship between vulvar condyloma, cervical HPV infection, and cervical epithelial abnormalities in an unvaccinated population may provide clinically relevant information for risk assessment and patient management.

Against this background, the present study was designed to investigate whether histopathologically confirmed vulvar condyloma is associated with concurrent cervical HPV-related disease. Specifically, we evaluated the relationship between vulvar condyloma and cervical cytological abnormalities, high-risk HPV genotype distribution, and cervical histopathological outcomes in women undergoing routine gynecological evaluation. By examining both virological and pathological findings within the same cohort, this study aimed to clarify the clinical significance of vulvar condyloma as a potential indicator of concurrent cervical HPV-related abnormalities in a population that has not yet benefited from widespread HPV vaccination.

## 2. Materials and Methods

This retrospective observational study was conducted at the Department of Obstetrics and Gynecology, Izmir City Hospital, Türkiye, between January 2025 and January 2026. The study aimed to investigate the association between histopathologically confirmed vulvar condyloma and concurrent cervical cytological abnormalities, high-risk HPV infection, and cervical histopathological findings in women undergoing routine gynecological evaluation.

Electronic medical records were reviewed consecutively to minimize selection bias. During the study period, all eligible women who underwent cervical cytology, HPV testing, vulvar examination, and colposcopic evaluation when clinically indicated were screened for inclusion. Clinical information, laboratory findings, cytological reports, HPV genotyping results, colposcopic findings, and histopathological diagnoses were extracted from the institutional electronic database.

Patients were categorized according to the presence or absence of vulvar condyloma. The condyloma-positive group consisted of women with clinically suspected vulvar lesions that were subsequently confirmed by histopathological examination after biopsy or excision. Women without clinical or pathological evidence of vulvar condyloma comprised the comparison group.

Patients were eligible if complete demographic information, cervical cytology, HPV testing, and follow-up records were available. Women with previous HPV vaccination, prior cervical cancer, previously documented abnormal cervical cytology requiring treatment, pregnancy, incomplete clinical records, or previously documented persistent HPV infection were excluded to reduce potential confounding related to prior cervical disease or persistent viral infection.

Cervical specimens were collected using the ThinPrep™ liquid-based cytology system according to routine clinical practice. Cytological evaluation was performed by experienced cytopathologists and classified according to the 2014 Bethesda System. Cytological diagnoses included negative for intraepithelial lesion or malignancy (NILM), atypical squamous cells of undetermined significance (ASC-US), low-grade squamous intraepithelial lesion (LSIL), atypical squamous cells—cannot exclude high-grade squamous intraepithelial lesion (ASC-H), and high-grade squamous intraepithelial lesion (HSIL).

High-risk HPV testing was performed using the Cobas^®^ 4800 HPV Test (Roche Molecular Systems, Pleasanton, CA, USA), a real-time polymerase chain reaction assay that individually detects HPV16 and HPV18 while simultaneously identifying a pooled group of 12 additional oncogenic HPV genotypes. The assay was performed according to the manufacturer’s recommendations within the institutional molecular microbiology laboratory. HPV16 and HPV18 positivity included isolated infections as well as co-infections, whereas “other high-risk HPV” represented pooled oncogenic genotypes without concurrent HPV16 or HPV18 detection.

Colposcopic examinations were performed by experienced gynecologic oncologists using standard colposcopic techniques after application of 3–5% acetic acid and Lugol’s iodine solution whenever clinically appropriate. The transformation zone was carefully evaluated, and biopsy specimens were obtained from acetowhite areas, abnormal vascular patterns, mosaicism, punctation, iodine-negative regions, or any lesion considered suspicious for cervical intraepithelial neoplasia according to routine clinical assessment.

Directed cervical biopsies were performed only when colposcopic findings indicated clinically relevant abnormalities. Histopathological examinations were carried out by experienced pathologists according to routine institutional practice. Cervical biopsy findings were classified as negative, CIN1, CIN2, or CIN3. For statistical analyses, CIN2 and CIN3 were combined and analyzed as CIN2+, consistent with internationally accepted clinically significant disease thresholds.

Women without biopsy indications continued routine follow-up according to institutional cervical screening protocols.

All statistical analyses were performed using IBM SPSS Statistics for Windows, Version 28.0 (IBM Corp., Armonk, NY, USA). Continuous variables were examined for normality using graphical methods and descriptive statistics. Normally distributed continuous variables were summarized as mean ± standard deviation (SD), whereas categorical variables were presented as absolute numbers and percentages.

Comparisons between patients with and without vulvar condyloma were performed using Student’s independent-samples t-test for continuous variables. Categorical variables were compared using Pearson’s chi-square test. Fisher’s exact test was applied whenever expected cell counts were insufficient for chi-square analysis.

Crude odds ratios (ORs) together with 95% confidence intervals (CIs) were calculated for HPV genotype distribution and cervical histopathological outcomes. Multivariable logistic regression analysis was subsequently performed to identify variables independently associated with vulvar condyloma. Variables considered clinically relevant or demonstrating statistical significance in univariate analyses were entered simultaneously into the multivariable model. Adjusted odds ratios (aORs) with corresponding 95% confidence intervals were reported.

Model assumptions were evaluated before regression analysis. Statistical significance was defined as a two-sided *p* value < 0.05.

## 3. Results

A total of 407 patients met the study inclusion criteria. The demographic and clinical characteristics of the study population according to vulvar condyloma status are summarized in Table 1. The mean age of the overall cohort was 32.1 ± 10.5 years, with an age range of 18–64 years. Vulvar condyloma was identified in 180 patients (44.2%), whereas 227 patients (55.8%) had no clinical or histopathological evidence of condyloma. Mean age was 32.5 ± 10.7 years in the condyloma-positive group and 31.7 ± 10.3 years in the condyloma-negative group (*p* = 0.465). Age-group distribution was 18–25 years in 61/180 (33.9%) and 79/227 (34.8%), 26–30 years in 35/180 (19.4%) and 45/227 (19.8%), and 31–64 years in 84/180 (46.7%) and 103/227 (45.4%) patients, respectively (*p* = 0.99).

Cervical cytological findings are shown in Table 1. NILM cytology was observed in 186/227 (81.9%) condyloma-negative patients and 114/180 (63.3%) condyloma-positive patients. ASC-US was observed in 29/227 (12.8%) and 42/180 (23.3%) patients, LSIL in 10/227 (4.4%) and 23/180 (12.8%) patients, ASC-H in 1/227 (0.4%) and 1/180 (0.6%) patients, and HSIL in 1/227 (0.4%) and 0/180 (0.0%) patients, respectively. The between-group comparison for cytology categories was significant (*p* < 0.001).

HPV test results are summarized in Table 1. HPV-negative results were observed in 141/227 (62.1%) condyloma-negative patients and 76/180 (42.2%) condyloma-positive patients. Any high-risk HPV positivity was observed in 86/227 (37.9%) and 104/180 (57.8%) patients, respectively (*p* < 0.001).

The distribution of high-risk HPV subtypes according to vulvar condyloma status is presented in Table 2. HPV16 and HPV18 positivity included both isolated infections and co-infections; therefore, the individual HPV16, HPV18, and other high-risk HPV counts are not expected to sum to the number of patients with any high-risk HPV positivity. Any high-risk HPV positivity was observed in 104/180 (57.8%) condyloma-positive patients and 86/227 (37.9%) condyloma-negative patients (OR 2.24, 95% CI 1.51–3.34, *p* < 0.001). HPV16 positivity was observed in 53/180 (29.4%) and 30/227 (13.2%) patients, respectively (OR 2.74, 95% CI 1.66–4.52, *p* < 0.001). HPV18 positivity was observed in 25/180 (13.9%) and 8/227 (3.5%) patients, respectively (OR 4.42, 95% CI 1.94–10.05, *p* < 0.001). Other high-risk HPV positivity without HPV16 or HPV18 was observed in 34/180 (18.9%) and 52/227 (22.9%) patients, respectively (OR 0.78, 95% CI 0.48–1.27, *p* = 0.330).

Multivariate logistic regression results are shown in Table 3. Adjusted ORs were 1.15 for age per 10 years (95% CI 0.95–1.40, *p* = 0.156), 2.30 for HPV16 positivity (95% CI 1.36–3.89, *p* = 0.002), 3.29 for HPV18 positivity (95% CI 1.40–7.73, *p* = 0.006), and 2.01 for abnormal cervical cytology (95% CI 1.24–3.25, *p* = 0.005).

High-grade cervical lesions are summarized in Table 4 as both an overall cohort analysis and a biopsy-restricted analysis. CIN2+ diagnosis was recorded in 22/407 (5.4%) patients in the study cohort. In the overall cohort, CIN2+ was recorded in 15/180 (8.3%) condyloma-positive patients and 7/227 (3.1%) condyloma-negative patients (OR 2.86, 95% CI 1.14–7.17, *p* = 0.026). No recorded CIN2+ diagnosis was observed in 165/180 (91.7%) and 220/227 (96.9%) patients, respectively.

In the biopsy-restricted analysis, 123 patients had cervical histopathological results available, including 82 condyloma-positive patients and 41 condyloma-negative patients. CIN2+ was identified in 15/82 (18.3%) condyloma-positive patients and 7/41 (17.1%) condyloma-negative patients (OR 1.09, 95% CI 0.41–2.92, *p* = 1.000). No CIN2+ was identified in 67/82 (81.7%) and 34/41 (82.9%) patients, respectively.

## 4. Discussion

The key observation of the present study is that a clinically visible vulvar HPV-related lesion identified a subgroup of women with a greater burden of cervical abnormalities. Rather than indicating that vulvar condyloma itself causes cervical dysplasia, the findings support a multifocal lower anogenital tract infection model in which external HPV-related lesions may coexist with cervical high-risk HPV infection and abnormal cervical cytology.

Persistent infection with oncogenic HPV genotypes, especially HPV16 and HPV18, is the principal mechanism underlying cervical carcinogenesis and the development of cervical intraepithelial neoplasia [11,12]. HPV infection is also responsible for a substantial proportion of infection-related cancers worldwide, highlighting its major global public health burden [13]. Although vulvar condyloma is classically linked to low-risk HPV types such as HPV6 and HPV11, previous studies have demonstrated that clinically visible genital warts may coexist with high-risk HPV genotypes [14,15]. This phenomenon likely reflects the multifocal nature of HPV infection within the lower anogenital tract, where the cervix, vulva, vagina, and anus may be simultaneously exposed to viral infection and persistence [16].

In the present study, abnormal cervical cytology was significantly more common among patients with vulvar condyloma. Similar findings have been reported in previous studies evaluating the relationship between genital warts and cervical epithelial abnormalities [17,18]. These observations support the hypothesis that visible vulvar HPV-related lesions may serve as a clinical marker of concurrent cervical HPV infection rather than an isolated benign manifestation.

An important finding of the present study was the independent association between vulvar condyloma and HPV16/18 positivity. Because HPV16 and HPV18 are the genotypes most strongly associated with cervical carcinogenesis, this finding may have clinical relevance in gynecological practice. The association remained significant after adjustment for age and abnormal cervical cytology, suggesting that visible vulvar disease may provide additional clinical information beyond cytology alone. Women presenting with vulvar condyloma may therefore benefit from careful confirmation that age-appropriate cervical cytology and HPV testing are up to date, even when cervical symptoms are absent.

These findings should also be considered in light of the emerging shift toward risk-based cervical assessment. Recent evidence suggests that viral load and genotype multiplicity may provide additional risk information beyond a binary HPV result, with higher viral loads and co-infections observed more frequently in high-grade CIN [7]. Similarly, integrating HPV genotyping and viral-load assessment with histopathology may improve diagnostic precision, particularly when morphological findings are equivocal [8]. Within this framework, visible vulvar condyloma should be regarded as a clinical cue to confirm that guideline-based cervical assessment is current, rather than as a standalone surrogate for cervical precancer or an indication for screening outside recommended intervals.

The CIN2+ findings require more cautious interpretation. Although recorded CIN2+ diagnoses were more frequent among patients with vulvar condyloma in the overall cohort, this association disappeared in the biopsy-restricted sensitivity analysis. This pattern suggests that the overall cohort result may partly reflect verification bias, because cervical biopsy was performed when clinically indicated rather than uniformly across all patients. Accordingly, the present data support vulvar condyloma as a marker of concurrent abnormal cervical cytology and high-risk HPV infection, but they do not establish a definitive independent association with histologically confirmed CIN2+.

An important characteristic of the present study population is that all participants were unvaccinated against HPV. Consequently, the observed prevalence of vulvar condyloma, high-risk HPV infection, and cervical cytological abnormalities reflects the natural epidemiology of HPV infection in the absence of vaccine-induced protection. This provides a valuable opportunity to evaluate the clinical relationship between vulvar condyloma and concurrent cervical HPV-related disease without the modifying effects of prophylactic vaccination.

The impact of HPV vaccination on HPV-related disease has been consistently demonstrated in countries with established immunization programs. National surveillance data from Australia have shown a rapid and sustained decline in the incidence of genital warts following implementation of a gender-neutral HPV vaccination strategy, highlighting the effectiveness of vaccination in reducing the burden of clinically apparent HPV infection [19]. Similarly, population-based cohort studies have reported a substantial reduction in the incidence of invasive cervical cancer among vaccinated women, confirming the long-term effectiveness of HPV vaccination against clinically significant cervical disease [20]. In addition, a comprehensive systematic review and meta-analysis demonstrated marked reductions in vaccine-type HPV infection, genital warts, and cervical intraepithelial neoplasia, together with evidence of herd protection in populations achieving high vaccine coverage [21].

The present findings should therefore be interpreted within the context of an unvaccinated population. As HPV vaccination becomes increasingly widespread, the epidemiological relationship between vulvar condyloma and concurrent cervical abnormalities may evolve because both the prevalence of genital warts and the circulation of vaccine-targeted oncogenic HPV genotypes are expected to decline. Future studies conducted in highly vaccinated populations will be important to determine whether vulvar condyloma retains similar clinical significance as a marker of concurrent cervical HPV-related disease or whether its predictive value changes in the post-vaccination era.

From a clinical perspective, these findings should not be interpreted as a recommendation for more frequent cervical cancer screening solely because external genital warts are present. Contemporary STI guidance indicates that, in the absence of other indications, external genital warts alone do not warrant intensified cervical screening; rather, the present results emphasize the importance of ensuring appropriate routine cervical cytology and HPV-based evaluation in women presenting with vulvar condyloma [22].

Several limitations should be considered when interpreting the findings of this study. First, the retrospective design is inherently susceptible to selection bias and limited our ability to account for all potential confounding variables. Although multivariable regression analysis was performed, information on several established risk factors for persistent HPV infection and cervical neoplasia, including smoking status, sexual behavior, parity, contraceptive use, and immunological conditions, was not consistently available in the medical records. Consequently, residual confounding cannot be completely excluded.

Second, this investigation was conducted at a single tertiary referral center. Patients referred to such institutions frequently represent a population with a higher pretest probability of HPV-related disease than women in routine screening settings. Therefore, the observed prevalence of high-risk HPV infection and cervical abnormalities may not be directly applicable to community-based or population-based screening cohorts.

Third, histopathological confirmation of cervical disease was available only for patients who underwent clinically indicated colposcopic biopsy. Women without biopsy indications were managed according to routine follow-up protocols based on cervical cytology and HPV testing. Consequently, histological verification was not uniformly available for the entire cohort, introducing the possibility of verification bias when evaluating CIN2+ outcomes. The biopsy-restricted sensitivity analysis was therefore included to provide a more conservative assessment of the observed association between vulvar condyloma and clinically significant cervical disease.

Finally, the relatively small number of CIN2+ cases limited the statistical power of subgroup analyses, particularly when evaluating histologically confirmed high-grade lesions. Larger multicenter studies with greater numbers of clinically significant outcomes would enable more precise estimation of effect sizes and facilitate more detailed analyses according to HPV genotype, cytological category, and histopathological diagnosis.

Despite these limitations, the present study has several important strengths. Vulvar condyloma was confirmed histopathologically in all affected patients, minimizing outcome misclassification at the level of the primary exposure. In addition, cervical cytology, HPV testing, and histopathological evaluation were performed within a standardized institutional diagnostic pathway, ensuring methodological consistency throughout the study period. The availability of detailed HPV genotype information also allowed separate evaluations of HPV16, HPV18, and other high-risk genotypes, providing clinically relevant insight into genotype-specific associations. Furthermore, inclusion of both overall cohort analyses and biopsy-restricted sensitivity analyses enhanced the robustness of the findings by allowing assessment of the potential influence of differential outcome verification. Collectively, these strengths support the reliability of the observed associations while emphasizing the need for confirmation in prospective studies.

## 5. Conclusions

In this cohort of unvaccinated women, vulvar condyloma was independently associated with abnormal cervical cytology, high-risk HPV infection, and HPV16/18 positivity. These findings support the concept that clinically apparent vulvar condyloma may represent a visible marker of concurrent HPV-related disease within the lower genital tract rather than an isolated benign condition.

Although women with vulvar condyloma demonstrated a higher frequency of recorded CIN2+ in the overall cohort, this association was not maintained in the biopsy-restricted sensitivity analysis, indicating that the relationship with histologically confirmed high-grade cervical disease should be interpreted with caution. Accordingly, the present findings support careful assessment of cervical cytology and HPV testing in women presenting with vulvar condyloma but do not justify intensified cervical cancer screening or altered management solely on the basis of the presence of external genital warts.

Prospective multicenter studies incorporating standardized colposcopic assessment, uniform histopathological verification, and longer follow-up are warranted to clarify the relationship between vulvar condyloma and clinically significant cervical neoplasia and to determine whether visible vulvar HPV-related lesions provide incremental value in contemporary risk-stratified cervical cancer prevention strategies.

## Figures and Tables

**Table 1:** Baseline Demographic and Clinical Characteristics According to Vulvar Condyloma Status.

Variable	Condyloma (−) *n* = 227	Condyloma (+) *n* = 180	*p* Value
**Age (mean ± SD)**	31.7 ± 10.3	32.5 ± 10.7	0.465
**Age group**			0.99
18–25 years	79 (34.8%)	61 (33.9%)	
26–30 years	45 (19.8%)	35 (19.4%)	
31–64 years	103 (45.4%)	84 (46.7%)	
**Cervical cytology**			<0.001
NILM	186 (81.9%)	114 (63.3%)	
ASC-US	29 (12.8%)	42 (23.3%)	
LSIL	10 (4.4%)	23 (12.8%)	
ASC-H	1 (0.4%)	1 (0.6%)	
HSIL	1 (0.4%)	0 (0.0%)	
**HPV test result**			<0.001
HPV negative	141 (62.1%)	76 (42.2%)	
Any high-risk HPV positive	86 (37.9%)	104 (57.8%)	

Abbreviations: NILM, negative for intraepithelial lesion or malignancy; ASC-US, atypical squamous cells of undetermined significance; ASC-H, atypical squamous cells—cannot exclude high-grade squamous intraepithelial lesion; LSIL, low-grade squamous intraepithelial lesion; HSIL, high-grade squamous intraepithelial lesion; HPV, human papillomavirus; SD, standard deviation.

**Table 2:** Distribution of High-Risk HPV Subtypes According to Vulvar Condyloma Status.

HPV Subtype/Marker	Condyloma (−) *n* = 227	Condyloma (+) *n* = 180	Crude OR (95% CI)	*p* Value
Any high-risk HPV positivity	86 (37.9%)	104 (57.8%)	2.24 (1.51–3.34)	<0.001
HPV16 positivity	30 (13.2%)	53 (29.4%)	2.74 (1.66–4.52)	<0.001
HPV18 positivity	8 (3.5%)	25 (13.9%)	4.42 (1.94–10.05)	<0.001
Other high-risk HPV only	52 (22.9%)	34 (18.9%)	0.78 (0.48–1.27)	0.330

Abbreviations: HPV, human papillomavirus; CI, confidence interval; OR, odds ratio. Note: HPV16 and HPV18 positivity are not mutually exclusive categories and include isolated infections and co-infections; therefore, subtype counts are not expected to sum to the number of patients with any high-risk HPV positivity. “Other high-risk HPV only” was defined as pooled high-risk HPV positivity without HPV16 or HPV18.

**Table 3:** Multivariate Logistic Regression Analysis of Variables Included in the Model for Vulvar Condyloma Status.

Variable	Adjusted OR	95% CI	*p* Value
Age, per 10 years	1.15	0.95–1.40	0.156
HPV16 positivity	2.30	1.36–3.89	0.002
HPV18 positivity	3.29	1.40–7.73	0.006
Abnormal cervical cytology	2.01	1.24–3.25	0.005

Abbreviations: OR, odds ratio; CI, confidence interval. Note: Outcome variable was vulvar condyloma. Model included all 407 patients. Abnormal cervical cytology was defined as ASC-US, LSIL, ASC-H, or HSIL.

**Table 4:** Association Between Vulvar Condyloma and High-Grade Cervical Lesions (CIN2+) in Overall and Biopsy-Restricted Analyses.

Outcome	Condyloma (−)	Condyloma (+)	OR (95% CI)	*p* Value
**Overall cohort (all included patients)**				
**No CIN2+ recorded**	220/227 (96.9%)	165/180 (91.7%)	Reference	
**CIN2+ recorded**	7/227 (3.1%)	15/180 (8.3%)	2.86 (1.14–7.17)	0.026
**Biopsy-restricted sensitivity analysis (patients with recorded cervical histopathology)**				
**No CIN2+**	34/41 (82.9%)	67/82 (81.7%)	Reference	
**CIN2+**	7/41 (17.1%)	15/82 (18.3%)	1.09 (0.41–2.92)	1.000

Abbreviations: CIN, cervical intraepithelial neoplasia; CIN2+, cervical intraepithelial neoplasia grade 2 or higher; OR, odds ratio; CI, confidence interval. The biopsy-restricted analysis included patients with available cervical histopathological results.

## Data Availability

The datasets generated and/or analyzed by this research are included in this paper and are publicly available in the Zenodo repository. https://zenodo.org/records/20334803 (accessed on 21 May 2026).

## Abbreviations

The following abbreviations are used in this manuscript:

ASC-H	Atypical Squamous Cells—Cannot Exclude High-Grade Squamous Intraepithelial Lesion
ASC-US	Atypical Squamous Cells of Undetermined Significance
CI	Confidence Interval
CIN	Cervical Intraepithelial Neoplasia
CIN2+	Cervical Intraepithelial Neoplasia Grade 2 or Higher
HPV	Human Papillomavirus
HR	High-Risk
HSIL	High-Grade Squamous Intraepithelial Lesion
LSIL	Low-Grade Squamous Intraepithelial Lesion
NILM	Negative for Intraepithelial Lesion or Malignancy
NS	Not Significant
OR	Odds Ratio
SD	Standard Deviation
